# Optimizing Catalyst Location within Nanostructured Photoelectrodes

**DOI:** 10.1021/acsami.5c11956

**Published:** 2025-08-19

**Authors:** Amin Farhadi, Theresa Bartschmid, Johannes Menath, Nicolas Vogel, Gilles R. Bourret

**Affiliations:** † Department of Chemistry and Physics of Materials, 27257 University of Salzburg, Jakob Haringerstraße 2a, Salzburg A-5020, Austria; ‡ Institute of Particle Technology, 9171 Friedrich-Alexander University Erlangen-Nürnberg, Cauerstrasse 4, Erlangen 91058, Germany

**Keywords:** silicon nanowires, water-splitting, metal-assisted chemical etching, photocathode, lithography

## Abstract

The influence of catalyst location within nanostructured photocathodes on conversion efficiency is reported. Highly uniform vertically aligned silicon nanowire (VA-SiNW) arrays prepared via colloidal lithography and metal-assisted chemical etching were used as a model-system photoelectrode for the hydrogen evolution reaction. Using three-dimensional electrochemical axial lithography (3DEAL), the Pt catalyst was located either at the top, center, or bottom of the SiNWs, or uniformly throughout the array. The SiNWs were spatioselectively passivated with an insulating coating to limit charge recombination at the silicon-electrolyte interface. Our results show that the most efficient catalyst position is the center of the SiNW, with an enhanced short-circuit photocurrent that is ca. 2 times and 2.9 times higher compared to top and uniform Pt distributions, respectively. This is attributed to better charge extraction in the nanowire center that mitigates losses due to charge recombination while optimizing light absorption within the SiNW, as revealed by three-dimensional electromagnetic simulations.

## Graphical abstract

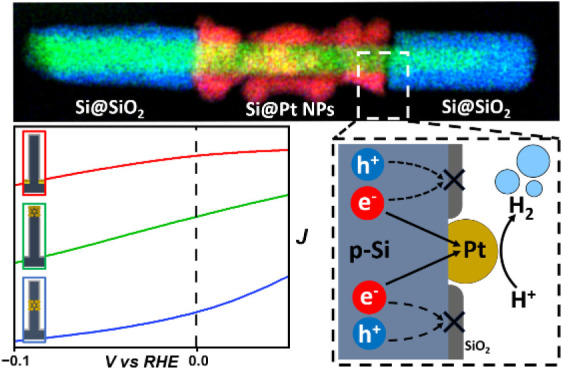

## 1 Introduction

Photoelectrochemical (PEC) hydrogen production has the potential to support the development of a sustainable and low-emission economy by using solar energy and water.^1–3^ Most semiconductors used for solar water-splitting suffer from insufficient light absorption, charge recombination, low catalytic activity, and/or (photo)­corrosion under operating conditions. Nanostructuring can address these issues by (i) shortening the travel distance required for photogenerated charges to reach the electrolyte, (ii) increasing light absorption thanks to a variety of optical resonances that can be excited within nanostructured semiconductors, (iii) decoupling light absorption from the charge separation process and (iv) combining passivating and catalytic layers with the semiconducting nanostructures to improve catalytic activity and electrode stability.^4–8^

For solar water-splitting, silicon is an appealing photoelectrode material because of its abundance, nontoxic nature, appropriate band gap for tandem cells, and the possibility to fully tune its optoelectronic properties using doping technologies developed by the microelectronics and photovoltaics industry. *P*-doped silicon (*p*-Si) is especially relevant for water-splitting photocathodes thanks to its appropriate conduction band edge energy and band-bending that are favorable for the hydrogen evolution reaction (HER).^7–10^ The ability to pattern silicon into defined nanostructures allows the introduction of additional functionalities that can be precisely tuned by varying the geometric features. Vertically aligned Si nanowire (VA-SiNW) arrays are a great example of nanostructured silicon, with strongly geometry-dependent optoelectronic properties,^6,11,12^ which have been utilized in a wide range of applications such as photonic systems and photodetectors,^11,13^ sensors,^14,15^ and solar conversion systems.^7,8^

In the context of HER, the high surface area of SiNWs allows for a high catalyst loading, and the decoupling of light absorption from the charge separation process,^6,8,16^ but can also increase recombination at the electrode–electrolyte interface, which may be mitigated by passivating the nanowire surface.^17^ For nanostructured photoelectrodes, such a passivation strategy requires the selective deposition of catalyst and insulating regions, which is synthetically challenging. Such an attempt was successfully demonstrated on microstructured Si photocathodes, composed of 4 μm-wide Si microwires, where the catalyst was deposited at the top of the wires.^18^ Despite this first demonstration, clear structure–property relations connecting the position of the catalyst with photoelectrocatalytic activities remain unknown. This is an overlooked issue, yet an essential one. Indeed, local catalyst activity can be significantly influenced by factors such as mass transport limitations, charge recombination, catalyst coverage, and defect distribution.^8,16,19–21^ Additionally, HER cocatalysts are often noble metals with significant optical losses across the solar emission spectrum, which can lead to pronounced decrease in conversion efficiencies in front-face illumination. Such losses can only be prevented if the catalyst amount, e.g., loading and location, is optimized, which is synthetically challenging on nanostructured photoelectrodes.

In this context, arrays of VA-SiNWs with diameters in the 150 nm range—25 times smaller than the 4 μm-wide microwires previously studied—are a relevant choice: Their lateral dimensions match those of nanostructured Si photoelectrodes, and more closely resemble the nanostructured designs commonly used for PEC systems, rather than microwires that are more specific. Additionally, these SiNWs strongly interact with light,^6,12^ and are expected to be optically sensitive to the presence of a metallic catalyst. One possible strategy to control the catalyst location within nanostructured silicon is the three-dimensional electrochemical axial lithography (3DEAL), which was developed using concepts pioneered by the on-wire lithography (OWL)^22,23^ and later on the coaxial lithography (COAL).^24,25^ 3DEAL can generate shells composed of plasmonic, catalytically active, or insulating SiO_2_ shells at defined locations within VA-SiNW arrays, with a spatial resolution down to the sub-10 nm range in both the axial and radial directions.^26,27^ In the context of PEC systems, 3DEAL was successfully used to spatioselectively deposit HER catalysts and insulating patches along SiNWs with diameters as small as 700 nm.^27^ However, patterning thinner SiNWs with a diameter of ca. 150 nm using 3DEAL has not yet been achieved.

Herein, we report the modification of 3DEAL to pattern arrays composed of short and thin VA-SiNWs (Figure 1). Different VA-SiNW photocathodes were prepared, where the Pt catalyst position was systematically varied. More precisely, our process allows fabricating nanostructured silicon substrates with the catalyst deposited at the top (Pt_top_), the center (Pt_center_), or at the bottom of the VA-SiNW arrays as a continuous film (Pt_film_), which we compare against a uniformly deposited Pt catalyst along the entire silicon surface (Pt_uniform_). We investigate in detail the PEC activity under white light irradiation as a function of catalyst location using the different photoelectrodes loaded with the same amount of catalyst. Additionally, the influence of optical losses in the metal catalyst was investigated via finite-difference time-domain (FDTD) three-dimensional electromagnetic simulations.

**Figure 1:**
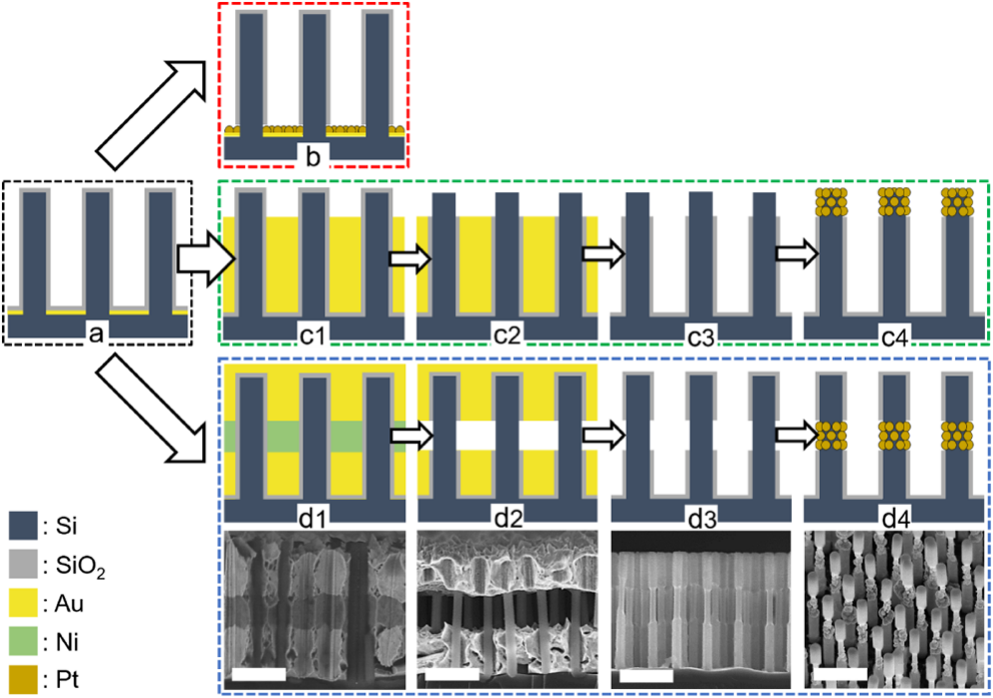
Schematic illustration of the modified 3DEAL process for the spatioselective deposition of Pt onto SiNW arrays in three different locations after (a) MACE and SiO_2_ passivation; (b) as a continuous Pt film on the SiNWs base; (c1–c4) as Pt particles at the top; (d1–d4) in the middle of the SiNWs. The corresponding SEM images of the SiNWs patterned with Pt in the middle are shown below each scheme (d1–d4). Scale bars: 1 μm. The materials composition is shown at the bottom left of the figure.

## 2 Results

### 2.1 Patterning of the Si Nanowire Arrays via 3DEAL

For our systematic investigation of the catalyst position, we first use colloidal lithography and metal-assisted chemical etching (MACE) in HF/H_2_O_2_ to synthesize well-ordered VA-SiNWs with the length, diameter, and pitch of 2114 ± 62 nm, 147 ± 7 nm, and 520 ± 29 nm, respectively (Figure S1).^28,29^ During MACE, the oxidizing agent is preferentially reduced at the surface of a nanostructured catalytic metal mesh, which injects holes into the silicon that oxidizes and dissolves as a soluble product in the presence of HF.^29^ After MACE, the catalytic metal film, here made of gold, is present at the bottom of the wires and can be dissolved if required. Under optimized conditions, MACE can reliably produce highly uniform VA-SiNW arrays.^28^ The array dimensions were chosen to obtain uniform VA-SiNWs that do not bundle due to capillary effects, and that can be patterned reliably via 3DEAL. Notably, these model systems differ from conventional black-silicon, prepared via MACE using Ag nanoparticles, and composed of highly dense and disordered arrays of long SiNWs (length > 10 μm), with highly inhomogeneous size distributions and morphologies.^30^ As a result, the overall reflectance of the well-defined VA-SiNW arrays synthesized in this work is higher than typical nanostructured Si photoelectrodes and our material exhibits a lower surface area for catalyst loading. However, our approach provides well-ordered and uniform arrays with defined dimensions and geometries, which is a prerequisite to establish fundamental structure–property relations connecting catalyst position with catalytic activity.

3DEAL is based on the electrodeposition of planar metal films that grow from the gold film present after MACE at the bottom of the VA-SiNW array.^26,27^ We revised the original 3DEAL sequence to improve the patterning process of our 150 nm-wide SiNWs (see Supporting Information Note I and Figure S2). In short: After MACE, a SiO_2_ conformal shell is grown preferentially around the VA-SiNW arrays via sol–gel deposition (Figure 1a).^6,26,27^ As demonstrated previously, planar metal films can be grown electrochemically from the gold film present after MACE. To deposit the Pt catalyst at a defined location in the center of the nanowire (Pt_center_, Figure 1d), a gold/nickel/gold trilayer film was sequentially grown (Figure 1d1). Selective Ni etching creates a gap between the two gold films (Figure 1d2). Subsequent KOH treatment dissolves the exposed silica shell in the gap region (Figure 1d3), providing nucleation sites to electrochemically deposit the catalyst layer at a specific axial location within the nanowire array (Figure 1d4). This KOH etch slightly reduces the SiNW diameter to approximately 120 nm (see Figure S4). In this work, Pt was electrochemically deposited on all samples at a constant current density of −0.3 mA.cm^–2^ for a duration of 189 s, thus providing similar catalyst loading on all samples with a porous nanocrystalline morphology consisting of Pt nanoparticles with average size of 3.5 ± 1 nm (Figures S5–S7). While the overall dimension of the large Pt deposits varies from sample to sample, the uniform nature of the primary particles forming the porous deposits is expected to result in a comparable catalytic activity and surface area.

To locate the catalyst at the top of the nanowires (Pt_top_, Figure 1c), a single gold film was deposited after MACE up to a defined location (Figure 1c1). KOH etching generates bare Si regions in the areas not protected by the Au film (Figure 1c2), which, after Au etching (Figure 1c3), are used to spatioselectively electrochemically deposit the Pt catalyst at the nanowire top (Figure 1c4). The catalyst can also be grown as a continuous Pt film located at the bottom of the SiNW arrays (Pt_film_) by electrodepositing Pt on top of the gold film used for MACE after encapsulating the SiNWs in a thin SiO_2_ layer (Figure 1b). Finally, bare VA-SiNW arrays can be coated uniformly with 36 ± 10 nm Pt porous particle structures by electrodepositing Pt directly after MACE, KOH etching and gold etching (Pt_uniform_).

### 2.2 Photoelectrochemical Results

Figure 2 shows the linear sweep voltammograms of the 5 defined models: bare VA-SiNW arrays, Pt_center_, Pt_top_, Pt_film_ and P_uniform_, under light irradiation (xenon lamp, AM 1.5 filter, 110 mW.cm^–2^), plotted against the reversible hydrogen electrode. The short-circuit current density *J*_*SC*_, e.g., photocurrent under zero applied bias, and the onset voltage *V*_*onset*_ at which the cathodic current density reaches −1 mA·cm^–2^ were used to compare the different samples. As shown in Figure 2 and Table 1, Pt_center_ consistently performs better than the other samples, with *J*_*SC*_ *=* 12.6 mA·cm^–2^ and *V*_*onset*_ = +0.25 V vs *RHE*. In comparison, Pt_top_ provides *J*_*SC*_ *=* 6.12 mA·cm^–2^ and *V*_*onset*_ = +0.24 V vs *RHE*, and Pt _uniform_ gives *J*_*SC*_ = 4.38 mA·cm^–2^ and *V*_*onset*_ = +0.18 V vs *RHE*. Additionally, when the Pt is present at the bottom of the SiNW as a film on top of the gold film, the short-circuit current decreases even further with *J*_*SC*_ = 2.00 mA·cm^–2^ while *V*_*onset*_ *=* + 0.16 V. As expected, the maximum applied bias photon-to-current efficiency (ABPE) of the samples follows the same trend: Pt_center_ > Pt_top_ > Pt_uniform_ > Pt_film_ (see Table S1 and Figure S8).

**Figure 2:**
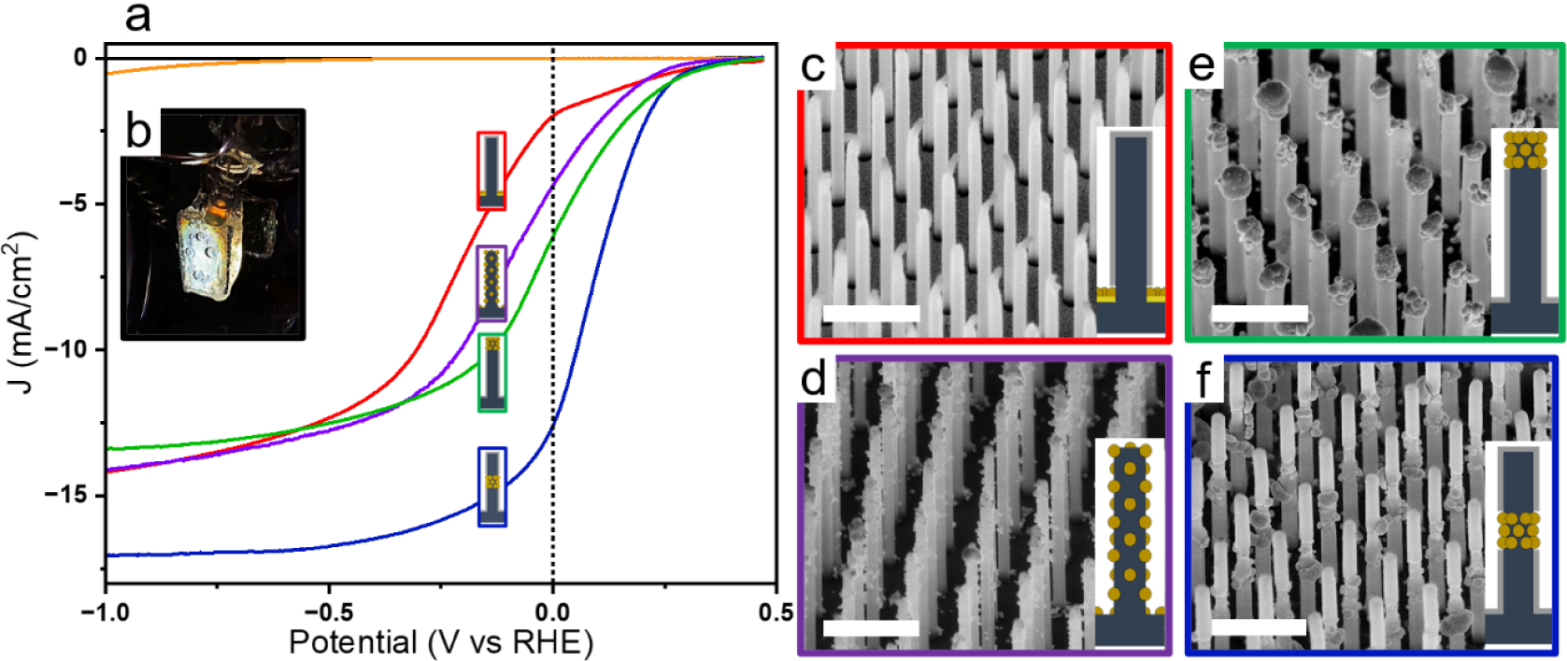
(a) Linear sweep voltammograms of five samples, measured at a scan rate of 100 mV/s. Under dark conditions, bare SiNWs (black solid line). Under light irradiation: Bare SiNWs (orange solid line), Pt_film_ (red solid line), Pt_uniform_ (purple solid line), Pt_top_ (green solid line), and Pt_center_ (solid blue line). (b) Photograph of a photocathode during photoelectrochemical measurement. (c–f) Tilted SEM micrographs of: (c) Pt_film_, (d) Pt_uniform_, (e) Pt_top_, and (f) Pt_center_. Scale bars: 1 μm. An SEM image of the bare VA-SiNW is shown in the SI (Figure S1).

**Table 1:** PEC Performance of the Samples Tested^a^

Sample	*J*_*SC*_ (mA·cm^–2^)	*V*_*onset*_ (V vs*RHE*)	Passivation layer
Bare SiNW	∼0	–1.15	No
Pt_uniform_	4.38	0.18	No
Pt_film_	2.00	0.16	Yes
Pt_top_	6.12	0.24	Yes
Pt_center_	12.6	0.25	Yes

a *J_SC_*: Short-Circuit Current Density, *V_onset_*: Onset Potential.

## 3 Discussion

Possible differences in Pt morphology and Si–Pt interface quality might explain these differences. However, the highly porous nature of the Pt deposits and the comparably uniform nature of the primary particles that form the deposits on all the SiNW samples investigated rules out any significant contribution of Pt particle size on PEC behavior. Additionally, we found that silicon surface treatment plays a marginal role and cannot by itself account for the large differences observed between the different samples. Indeed, the use of different etchant sequence and composition (KI/I_2_, KOH and/or HF) did not significantly affect the PEC behavior of the reference Pt_uniform_ sample, with *J*_*SC*_ comprised between ca. 3.52 mA·cm^–2^ and 4.9 mA·cm^–2^ for all samples tested (see Figure S9). Thus, other factors must contribute to the different PEC responses observed.

### 3.1 Influence of Nanowire Passivation

It is known that charge recombination is much higher at the surface of semiconductor nanowires. In fact, a decrease in minority charge carrier diffusion length by ca. 1000 times was reported on passivated SiNWs synthesized via MACE, compared to the bulk.^31^ Recombination can therefore become a serious issue even when using electronic grade single-crystalline silicon to prepare nanostructured surfaces. Thus, we attribute the higher short-circuit photocurrent measured on the passivated Pt_center_ and Pt_top_ samples, compared to the unpassivated Pt_uniform_, to reduced charge recombination at the silicon-electrolyte interface (Figure 3). Additionally, the superior performance of Pt_center_ compared to Pt_top_ may be attributed to the shorter distance that electrons photogenerated in the SiNWs must travel to reach the Pt catalyst when it is positioned at the center rather than at the top (Figure 3a,b): Even with appropriate passivation, recombination still plays a significant role, which is consistent with lifetime measurements performed on passivated SiNWs prepared via MACE.^31^ The fact that Pt_film_ exhibits such a low *J*_*SC*_ suggests that the charges photogenerated within the SiNWs could not efficiently reach the Pt catalyst located at the bottom of the SiNWs. This further emphasizes the importance of charge recombination, even within passivated SiNWs. It also suggests that the charges generated in the bulk of Pt_film_, i.e., the Si wafer beneath where recombination should be minimal, do not significantly participate in photocurrent generation.

**Figure 3:**
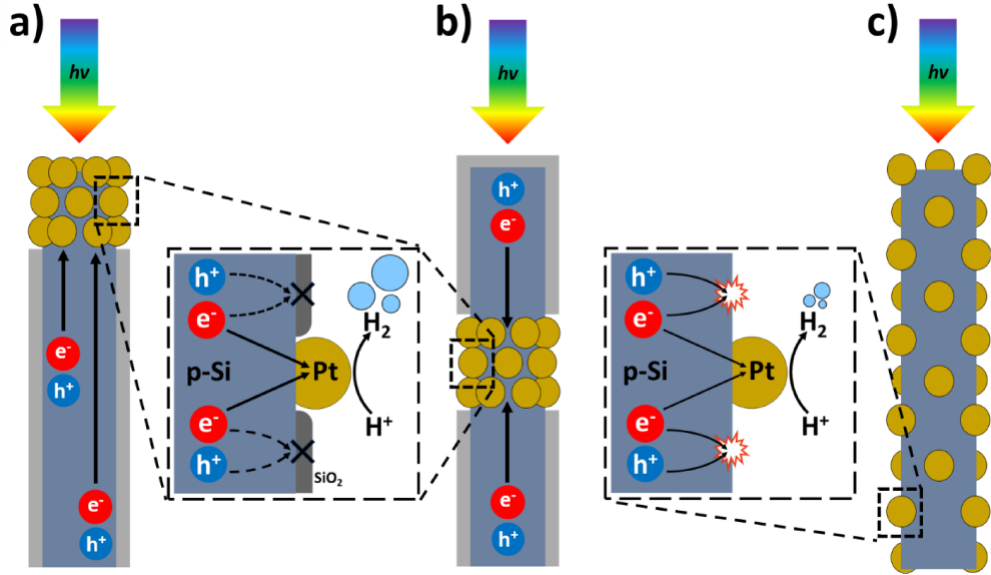
Schematic presentation of photoinduced charge dynamics in Pt-decorated SiNWs. Locating Pt at the top (a) and Pt at the center (b), and selective SiNW passivation, limits charge recombination inside the SiNW and at Si/electrolyte interface. In contrast, uniform Pt nanoparticle decoration without the passivating layer results in increased recombination and consequently reduced hydrogen evolution (c).

### 3.2 Optical Characterization

To investigate these differences, we estimated the amount of light absorbed in the silicon wafer for the different samples. Total reflectance measurements show that the samples coated with nanoscale Pt, i.e., Pt_center_, Pt_top_ and Pt_uniform_, all have a reduced reflectance in the 300–1200 nm range compared to the bare uncoated VA-SiNW arrays (Figure 4) because of the optical losses incurred by the Pt (see Figure S10 showing the decreased reflectance resulting from electrochemically deposited Pt nanoparticles on flat Si). This is consistent with our previous results on VA-SiNW arrays uniformly coated with AuNPs.^14^ However, when a Pt film is present instead (Pt_film_), the integrated reflectance losses are slightly higher (Figure 4b). Because flat silicon, SiNWs and Pt all strongly interact with light, i.e., absorb and reflect light, disentangling the negative contribution of the Pt catalyst on light absorption in the Si is not straightforward.

**Figure 4:**
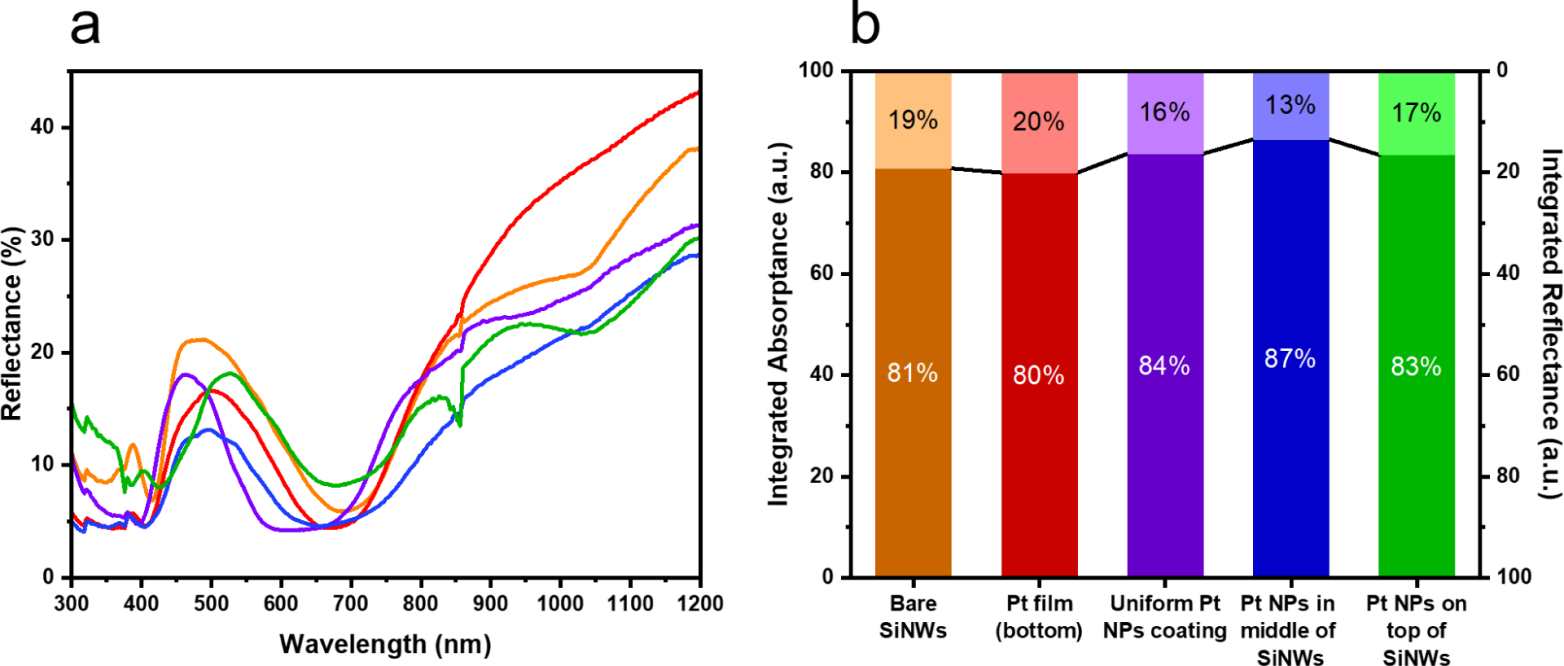
(a) Experimental total reflectance spectra within the 300–1200 nm wavelength range for: bare SiNWs (orange), Pt_film_ (red), Pt_uniform_ (purple), Pt_top_ (green), Pt_center_ (blue). (b) Corresponding normalized integrated values of absorptance (lower stacks) and reflectance (upper stacks), same color scheme as in (a).

### 3.3 Optical Simulations

To understand the optical consequences of coating SiNW arrays with a Pt catalyst, we performed three-dimensional electromagnetic simulations using the FDTD method (Figure 5).^12,23^ The total volume of Pt was identical for all simulations unless stated otherwise and chosen to reflect the experimental conditions, determined by the charge density used during the Pt deposition, assuming a 100% Faradaic reduction efficiency of the Pt (IV) ions (see the simulation section 5.11 in Materials and Methods for more details). Different Pt catalyst locations, morphologies, e.g., nanoparticle(s) or shell, and dimensions were compared, summarized in Table 2 and presented in Figure S12. We find that bare VA-SiNWs, i.e., without the Pt catalyst layer, absorb ca. 32% of the incoming light in the 300 – 1200 nm range, which is ca. 3 times more than the same volume of bulk silicon, corroborating previous work.^12^ This increased light absorption is due to the excitation of guided modes that strongly absorb around 400 nm and in the 600–800 nm range (Figures S13 and S14). We assessed the influence of the Pt catalyst by comparing the local integrated absorbed power and light absorption at defined locations within the arrays, i.e., within the SiNW, the Pt catalyst, and in the supporting Si wafer beneath (Figure 5). As expected, Pt absorbs light in the 300 – 1200 nm, resulting in optical losses ranging from 7% to 32% that depend on the position of Pt within the nanostructure array. Absorption losses decrease in the following order: Pt_film_ > Pt_center_ > Pt_top_ > Pt_uniform_ (Figure 5h), with especially large absorption for Pt_film_. Combined with the much higher reflectance of Pt_film_ (Figure 5h), it becomes clear that the presence of the Pt film significantly limits the penetration of light into the Si beneath: Only ca. 3% of the light reaches bulk Si located under the Au/Pt film, compared to 40–50% for all the other samples (Figure 5h). Such a drop in light transmission when having a continuous Pt film significantly prevents efficient light absorption, which most likely explains the low *J*_*SC*_ measured on the Pt_film_ sample. The situation is different when looking at the light absorbed inside the SiNWs where Pt_film_ shows a slightly increased light absorption compared to the bare VA-SiNW array (Figure 5g), which might be attributed to plasmonic effects and metallic back-reflection of the platinum hole array.^32^ However, this has only a minor influence on photocurrent generation, most likely because of suboptimal transport of the charges through the SiNW to reach the Pt film catalyst.

**Figure 5:**
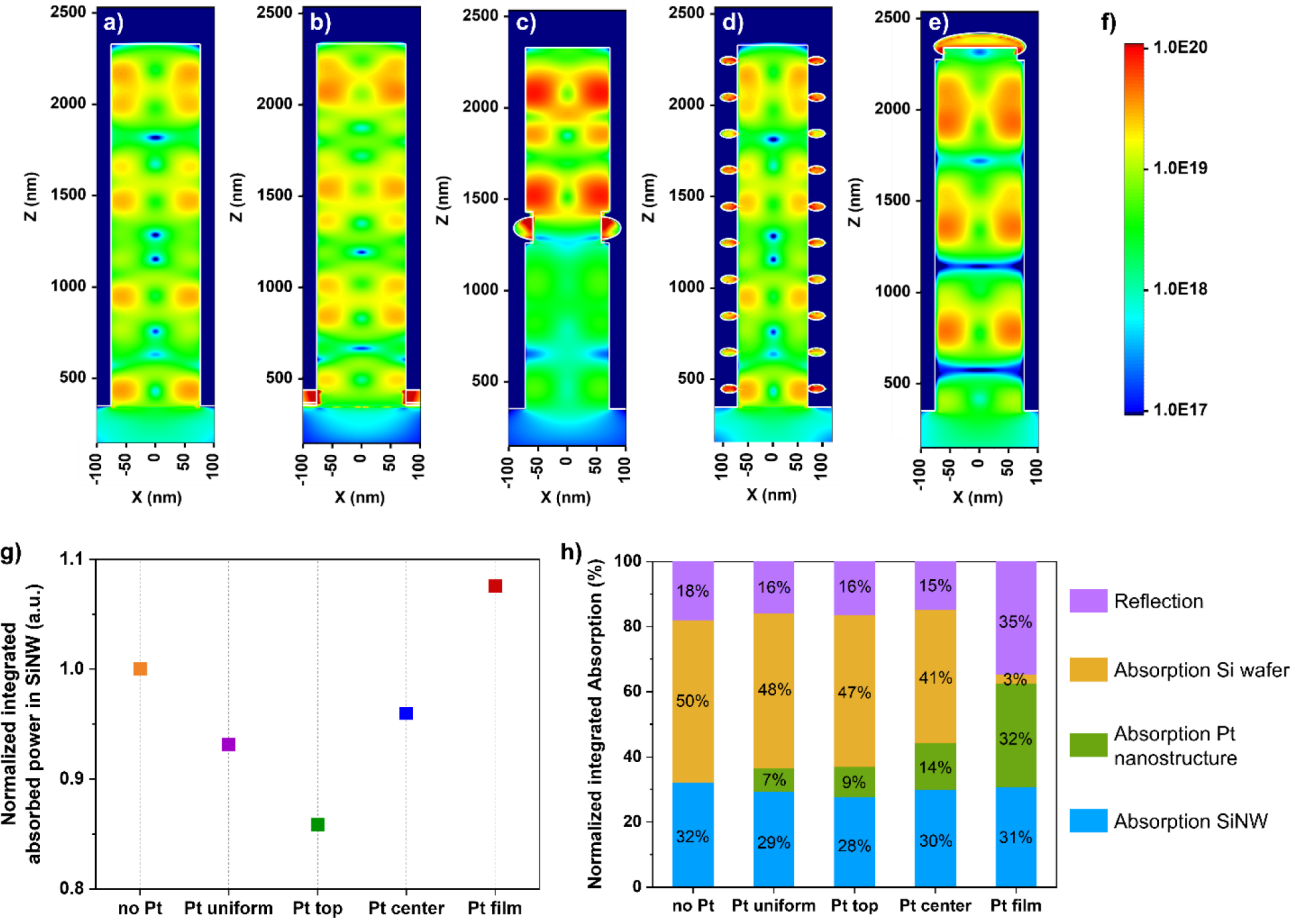
(a–e) Maps of absorbed power at a wavelength of 700 nm for bare SiNWs (a), Pt_film_ (b), Pt_center_ (c), Pt_uniform_ (d) and Pt_top_ (e). (f) Color scale of the absorbed power maps shown in (a–e). (g) Normalized integrated absorbed power in the SiNW for the different samples. (h) Normalized integrated reflection and absorption in the SiNW, the Pt nanostructure and the Si wafer for the different samples. (g, h) Values integrated in the range from 300 to 1200 nm.

**Table 2:** Simulations: Pt Catalyst Dimension and Morphology, and Corresponding Absorption Losses in the SiNW

Sample name	Pt morphology	Pt dimensions^a^	Absorption losses in the SiNW due to Pt^b^
Pt_uniform_	40 NPs	*d* = 34 nm	*–7%*
Pt_film_	Flat film	70 nm thick Pt film on top of a 20 nm thick gold film	+8%
Pt_top_	1 NP	*d* = 146 nm	*–14%*
Pt_center_	1 shell	*d*_*i*_ = 120 nm and *d*_*o*_ = 174 nm	*–4%*

a *d*: diameter of the NPs. *d*_*i*_ and *d*_*o*_: inner and outer diameter of the Pt shell, respectively.

b Absorption losses compared to the bare SiNWs, i.e., no Pt, calculated with the ratio (in%) = (1 *–* A_SiNW with Pt_/A_bare SiNW_) × 100, where A_SiNW with Pt_ and A_bare SiNW_ are the integrated absorbed powers in the SiNWs of the samples coated with Pt, and of the bare SiNW, respectively. For each sample, the absorbed power was integrated over the SiNW volume and the 300–1200 nm range, and is plotted in Figure 5g.

For the three other samples, we can relate the absorption losses due to the Pt catalyst to the amount of light reaching the SiNWs, with the greatest loss observed for Pt_top_, where the SiNW absorbs 14% less than the uncoated bare SiNW (Figure 5g). This is followed by Pt_uniform_ and Pt_center_, with absorption losses of 7% and 4%, respectively (Figure 5g): Locating the catalyst at the center of the SiNW optimizes light absorption within the SiNW, offering another advantage over Pt_uniform_ and Pt_top_. Additionally, an antenna effect is visible on the sample Pt_center_: The top half of the SiNW located above the Pt catalyst ring (Figure 5c and Figure S14) absorbs ca. 2.35 times more than the bottom half, which is a relative increase of ca. 30% compared to the bare SiNW, where absorption is ca. 1.80 times higher in the top half. This additional factor might also contribute to the large photocurrent generated on Pt_center_.

In summary, our simulations demonstrate the following optical effects on the PEC performance: (i) a continuous Pt film leads to large losses that prevents absorption in the Si wafer beneath, (ii) locating the Pt catalyst in the center rather at the top of the wires or uniformly distributed at the Si surface, optimizes light absorption within the SiNW, and (iii) the Pt shell present in the center of the SiNW acts as an antenna that concentrates light absorption in the top half of the nanowire. Our SiNW arrays are supported on a bulk Si wafer substrate, which significantly absorbs light and thus takes an active part in the PEC response. However, many PEC systems are based on nanostructured absorbers, often with a nanowire-like shape, supported on an optically inactive substrate used for charge extraction. Our simulation results are thus relevant for such photoelectrodes where light is only absorbed within the semiconductor nanowire.

## 4 Conclusion

Photoelectrochemical energy conversion is important, in particular for green hydrogen production, but requires expensive and rare metal catalysts. To optimize the use of these catalyst particles requires understanding the optoelectronic and photocatalytic properties of such hybrid systems. Here, we use the 3DEAL method to fabricate defined nanostructured model electrodes that allows controlling the catalyst position. This enables us to systematically study structure–property relations to determine how the catalyst position influences PEC conversion. We find that placing the Pt catalyst at the SiNW center outperforms all other geometries, with an enhanced short-circuit photocurrent that is ca. 2 times and 2.9 times higher compared to arrays coated with a Pt catalyst on top of each wire, or uniformly deposited throughout the array, respectively. This large increase in photocurrent demonstrates that appropriately locating the catalyst within nanostructured photoelectrodes is key to optimize catalyst loading, which provides an additional handle to increase further PEC conversion efficiency. FDTD simulations also provide an understanding of the origin and nature of the optical losses, showing that the catalyst location affects both the absolute and relative light absorption inside the nanowire and in the supporting substrate, with direct consequences for charge photogeneration and resulting photocurrent.

## 5 Materials and Methods

### 5.1 Materials

All chemicals and solutions cataloged here were used without further processing, unless stated otherwise. Acetone (99%), ethanol (96%), 2-Propanol (≥98%), calcium chloride (90–98%, Technical), hydrogen peroxide (30%), hydrofluoric acid (40%), nitric acid (65%), sulfuric acid (95–97%) and tetraethyl orthosilicate (TEOS, 99%) were acquired from VWR, Europe. Iodine (99.8%), potassium iodide (99.99%), gallium–indium eutectic (>99.99%, trace metals basis), nickel­(II)­sulfate hexahydrate (98%, ACS grade), Chloroplatinic acid hydrate (≥37.5% Pt basis), potassium hydroxide (pellets GR for analysis) and disodium phosphate (98–102%) were purchased from Sigma-Aldrich. Boric acid (99+%), were purchased from Alfa Aesar, USA. The water used was double deionized using a Milli-Q system with a resistivity of 18 MΩ·cm. Commercially available gold plating solution (Orotemp 24 Rack for Au) was purchased from Technic Inc. P-type silicon wafer (B-doped, 100 mm, ⟨100⟩, resistivity 1–30 Ω·cm, thickness: 525 ± 25 μm) were purchased from Si-Mat, Germany. Conducting silver paste ACHESON G3692 was purchased from PLANO GmbH, Germany. Conductive silver epoxy (CW2400) and electrically insulating epoxy (PLUS SOFORTFEST) were purchased from Chemtronics and UHU respectively. For the fabrication of SiO_2_@PNiPAm core–shell particles used as a colloidal template, ethanol (Sigma-Aldrich, 99.9%), N,N′- Methylenebis­(acrylamide) (BIS; 99%, Sigma-Aldrich), ammonium persulfate (APS, Sigma-Aldrich, 98%), hexane (≥99%), tetraethyl orthosilicate (98%), ammonium hydroxide solution (28–30% NH_3_ basis), (trimethoxysilyl)­propyl methacrylate (MPS, 98%), fluorescein isothiocyanate isomer I (FITC, >90%), and (3-aminopropyl)­triethoxysilane (APTES, >98%) were purchased from Sigma-Aldrich and were used in their original condition. *N*-Isopropylacrylamide (NIPAM, 97%, Sigma-Aldrich) was purified before usage by recrystallization from hexane (95%, Sigma-Aldrich).

### 5.2 Synthesis of the SiO_2_@PNIPAM Core–Shell Particles

Silica-poly­(*N*-isopropylacrylamide) (PNIPAM) core–shell particles were synthesized, self-assembled at the air/water interface and used as a mask is described in detail in the literature.^33,34^ In short silica cores with the diameter of 160 nm (±10 nm) were synthesized via the Stöber process. The silica nanoparticles then were functionalized with MPS by stirring at room temperature for a minimum of 24 h, followed by a 1-h boiling step to ensure complete functionalization. In the subsequent step, the particles underwent purification via three cycles of centrifugation and redispersion in ethanol and Milli-Q water. A PNIPAM microgel shell then was polymerized on the silica nanoparticles using surfactant-free precipitation polymerization with 2.5 mol % BIS as a cross-linker and APS as an initiator. Eventually, the as prepared core–shell nanoparticles were purified via centrifugation and redispersion with Milli-Q water.

### 5.3 Colloidal Monolayer Preparation

To generate colloidal monolayers as templates for MACE, we used SiO_2_–PNIPAM core–shell particles that were self-assembled at the air/water interface by a Langmuir–Blodgett trough (KSVNIMA) as described in detail in the literature.^14,33,34^ The p-type silicon wafer was cut into rectangular pieces (ca. 2.5 × 8 cm^2^) and then cleaned sequentially by ultrasonication in acetone and 2-propanol for 5 min each. A final 7 min oxygen plasma etch (Diener, Germany) was performed to ensure hydrophilicity. Silicon pieces were mounted at a 90° angle and dipped in a Langmuir–Blodgett trough filled with Milli-Q water. A 1:1 mixture of ethanol and core–shell particles was spread at the air–water interface. After 10 min of equilibration, the barriers were compressed to a constant surface pressure of 30.5 mN/m. The silicon substrates were then vertically lifted from the trough, resulting in the self-assembly of the core–shell particles into a well-ordered hexagonal arrangement on the substrate surface.

### 5.4 Fabrication of the SiNWs

SiNWs arrays were fabricated via a combination of colloidal lithography^33,34^ and metal-assisted chemical etching (MACE)^28^ previously developed in our group. In short, the Si substrates coated with SiO_2_@PNIPAM core–shell particles underwent an oxygen plasma treatment (50 W, 20 sccm, 12 min) in order to completely etch their polymeric shell. An adhesive Ti layer was sputtered afterward onto the substrates for 5 s at 100 W using a Clustex 100 M sputtering system by Leybold Optics. Subsequently, Au was sputtered for 200 s at 40 mA (Cressington Sputter Coater 108 auto). The silica particles were pulled-off using an adhesive tape yielding a gold nanomesh which acts as a mask during further MACE step. The Si/Au mesh substrates were etched in a MACE solution (10 mL H_2_O, 10 mL HF, 1 mL H_2_O_2_) for 5 min, followed by three rinses in Milli-Q water. The samples were subsequently etched in a 20 mL H_2_O and 4 mL HF mixture for 5 min to remove residual porous silica formed on the surface of the SiNWs. After three additional water rinses and a final ethanol rinse, the samples were dried in air.

### 5.5 Spatioselective Deposition of the HER Co-Catalysts via 3DEAL

To precisely locate the HER cocatalyst at the middle or top of the SiNWs, a modified three-dimensional electrochemical axial lithography (3DEAL)^26^ process was employed. In the first step, the SiNWs were encapsulated with a thin SiO_2_ insulating layer (ca. 10 nm) using a sol–gel method^27^ to prevent parasitic metal deposition during subsequent electrodeposition steps. Successive metal deposition was carried out using a Keithley 2400 Sourcemeter in a three-electrode electrochemical cell. A Pt mesh served as the counter electrode, an Ag/AgCl electrode as the reference electrode, and the Au film at the bottom of the SiNWs array (remaining after MACE) acted as the working electrode. The steps of the 3DEAL process are schematically illustrated in Figure 1. Depending on the desired location of the HER cocatalyst (either at the middle or top of the SiNWs), one or two distinct metallic layers were electrodeposited. In order to locate the HER cocatalyst at the middle of the wires (Figure 1d), a gold layer was initially deposited at −0.95 V for ca.1300 s minutes from an Orteorack solution, followed by nickel deposition at −0.9 V for ca. 800 s minutes using a custom-made solution (0.2 M NiSO_4_·6H_2_O + 0.15 M H_3_BO_3_). Finally, a top gold layer was deposited (ca. 2500 s) to fully cover the SiNWs. The thicknesses of the initial gold and nickel films respectively determine the height and width of the SiNW section that will be decorated with the catalyst. After metal deposition, the sandwiched Ni film was selectively etched in a 1:1 H_2_O:HNO_3_ solution for 18–24 h. Subsequently, the sample was immersed in a 20 wt % KOH aqueous solution for 12–18 min to selectively etch the silica shell in the regions previously covered by Ni. In contrast, to locate the HER cocatalyst at the top of the SiNWs, only a single gold layer was deposited (−0.95 V, 3000 s), leaving the top of the wires exposed. This sample was then immersed in a 20 wt % KOH aqueous solution for 8–10 min to etch the silica shell at the top of wires. After removal of silica shell in the desired parts of wires, the gold film was removed using a mixture of 85% H_2_O, 10 wt % KI, 5 wt % I_2_. Now loading HER cocatalyst can be realized at the regions free of insulating SiO_2_ shell. Pt was used as the HER cocatalyst in this work. All samples were loaded with the same amount of Pt, which was electrodeposited at a constant current of −0.3 mA for 3 min. To achieve uniform Pt NP decoration on the SiNWs, the native silica shell surrounding the entire SiNWs was first etched away using KOH solution for ca. 30 s, which was sufficient to dissolve the native SiO_2_. This is supported by the observation of bubbles being generated at the surface of the entire sample, which indicates the oxidation and dissolution of silicon by KOH. Subsequently, the initial gold base film (remaining from MACE) was first removed using the KI/I_2_ solution. Finally, Pt was electrodeposited onto the bare SiNWs surface. To deposit a Pt film at the base of the SiNWs, Pt was directly electrodeposited onto the remaining gold base film from the MACE process under standard electrochemical conditions.

To investigate parasitic absorption by Pt on the Si surface, a control experiment was conducted. A Si substrate, from the same wafer as the other samples, was cut and treated with 20 wt % KOH aqueous solution for 3 h to remove surface oxides. Subsequently, Pt nanoparticles were electrodeposited onto the Si substrate at a constant current of 0.3 mA for 3 min. Reflectance spectroscopy was then performed in the 300–1200 nm range.

### 5.6 Photocathode Preparation

A glass cutter was used to scratch the backside of a silicon (Si) substrate. A Ga–In eutectic mixture was then applied to the scratched area. Copper wires, inserted into a glass tube, were adhered to the substrate’s backside using a silver conductive epoxy and left to cure for 18 h. Subsequently, the photoelectrode’s back, sides, and front (excluding the Pt-decorated area) were covered with electrically insulating epoxy and dried for 18 h prior to photoelectrochemical measurements (Figure S11). The front side of the photocathodes was photographed, and the active surface area was measured using ImageJ.

### 5.7 Photoelectrochemical Measurements

Photoelectrochemical measurements were conducted using an Autolab PGSTAT302N potentiostat in a three-electrode configuration with a Pt wire counter electrode and a Ag/AgCl (3 M NaCl) reference electrode. The irradiation source composed of a 300 W Xe lampe (LotOriel) equipped with a water-cooled aperture and an Air Mass AM1.5G filter (Newport) delivering an intensity of approximately 110 mW·cm^–2^ (calibrated with an International Light IL1400A photometer) at the photocathode front-face. The electrolyte used was an aqueous 0.1 M H_2_SO_4_ solution (pH = 1) and purged with N_2_ for 1 h prior the test. Prior to the measurements, all photocathodes were cycled eight times in the dark between +0.2 and −1.6 V (vs reference electrode) until the current stabilized. Subsequently, linear sweep voltammograms were recorded at a scan rate of 100 mV/s. In order to process the data, the photocathodes potentials vs Ag/AgCl electrode (*E*_*Ag/AgCl*_) were converted to the reversible hydrogen electrode scale (*E*_*RHE*_) as using eq 1:

1 $$E_{RHE}=E_{Ag/AgCl}+0.059\times pH+{E^{{}^{\circ}}}_{Ag/AgClvsSHE}$$

where *E°*_*Ag/AgCl vs SHE*_ is the potential of the Ag/AgCl reference electrode vs standard hydrogen electrode (SHE), and for an Ag/AgCl electrode stored in a 3 M NaCl solution is 0.209 V. Therefore, the overall shift in the potentials for the RHE was calculated using *E*_*RHE*_ = *E*_*Ag/AgCl*_ + 0.268 V.

### 5.8 Total Reflectance UV–Vis Spectroscopy

Total Reflectance UV–vis spectra were acquired using PerkinElmer Lambda 1050 equipped with a 150 mm integrating sphere, collecting both the diffuse and the specular reflectance. A 5 mm circular aperture was used to select the area of interest. The light beam was aligned with the aperture using a white Spectralon reference, and baseline correction was performed. Reflectance spectra were recorded between 200 and 1200 at 2 nm step.

### 5.9 Electron Microscopy

Scanning electron microscopy (SEM) images were acquired using a Zeiss Ultra Plus 55. Imaging was performed at an accelerating voltage of 5 kV, with an InLens secondary electron (SE) and an angle selective backscattered electron (AsB) detectors and a working distance of 4 mm. Scanning transmission electron microscope (STEM) images were obtained using a cold field emission gun JEOL F200 STEM/TEM operated at 200 kV with a probe diameter of 0.16 nm and a probe current of 0.1 nA. Energy-dispersive X-ray spectroscopy (EDX) mapping was carried out using a large windowless JEOL Centurio EDX detector (100 mm^2^, 0.97 srad, energy resolution <133 eV).

### 5.10 Electromagnetic FDTD Simulations

Electromagnetic FDTD simulations were performed in the software Ansys Lumerical FDTD by Ansys Inc. (Canonsburg, USA)^35,36^ Dielectric functions of silicon, silicon dioxide, gold and platinum were used as available in the software to depict the optical response of the respective materials. Antisymmetric, symmetric and perfectly matched layers (PML) boundary conditions were used in x, y, and z directions, respectively.

The geometrical parameters of the SiNW arrays were a wire diameter of 150 nm, a wire length of 2000 nm and a center-to-center distance (pitch) of 520 nm. A volume of 808 × 10^3^ nm^3^ Pt per SiNW was used.

### 5.11 Calculation of Pt Volume per SiNW

The total mass of deposited platinum (Pt) during electrodeposition was determined by first calculating the total charge transferred (Q = 0.3 mA × 189 s = 0.0567 C). Subsequently, the number of transferred electrons was derived from the charge: *n*_*e*_ = Q/e = 3.54 × 10^17^, where e is the elementary charge. Considering a four-electron reduction for each Pt­(IV) ions, the number of deposited Pt atoms was calculated as *n*_*e*_/4 = 8.85 × 10^16^. The final Pt mass was then determined using the number of Pt atoms, Avogadro’s number, and the molar mass of Pt. The cumulative deposited Pt mass on a 0.44 cm^2^ sample area was 29 μg.

The number of SiNWs within a 0.44 cm^2^ sample was determined assuming a perfect hexagonal array. The total deposition area was divided by the area of a single hexagon, i.e., 8.7685 × 10^–10^ cm^2^, to find the number of hexagons. Since each hexagon contains three SiNWs, the total number of SiNWs in the deposition area was *n*_*wires*_ = 1.5054 × 10^9^.

The Pt volume per SiNW was ultimately determined from its mass (29 μg) and density (21.45 g/cm^3^). Here, it was estimated from SEM images that ca. 90% of the Pt end up on the SiNWs whereas ca. 10% are parasitically grown at the flat Si bottom of the SiNW array, yielding an estimated volume of Pt per SiNW of 808290 nm^3^.

### 5.12 Dimensions of the Heterostructured Arrays

For the samples Pt_center, shell_, Pt_center, 40 NPs_, Pt_top, 60 nm_, Pt_top, 73 nm_, and Pt_top, 110 nm_ the SiNWs had a diameter of 120 nm at the location where the PtNP was placed. Those values were obtained from measuring the distances on SEM images of the investigated samples. The thickness of the silicon substrates carrying the SiNWs was set to 1341 nm. Both, the silicon substrate as well as the SiNWs were covered with a 2 nm thin layer of SiO_2_ (included in the dimensions given above) to mimic the native oxide layer that is naturally growing on top of Si surfaces in air. The simulations were performed in vacuum (*n* = 1). The mesh size was set to 4 nm around the SiNWs.

Linearly polarized light from a plane wave source was used for illumination of the sample in a wavelength range from 300 to 1200 nm. One frequency domain power monitor was placed behind the source and another one 130 nm below the bulk silicon surface to get information on the reflectance and transmittance spectra of the Pt-SiNW array, respectively. Shown UV–vis data were smoothened.

The advanced absorption monitor was set around the SiNWs in order to extract the absorbed power depending on the type of material (silicon or platinum). The resulting absorbed power spectra for the SiNWs were integrated across the 300 nm–1200 nm range, normalized and compared in Figure 5g.

In order to extract the relative contributions of the absorption in the bulk Si wafer, the Pt and the SiNWs to the overall sample absorption (see Figure 5h), the reflectance and transmittance monitors (2D) and absorbed power monitors (3D) were used. The 2D transmittance monitor was located 130 nm below the Si surface to calculate the absorptance spectrum (A_wafer_) of the bulk Si wafer, equal to the transmittance spectrum T_SiNWs+PtNPs_ behind the SiNWs coated with PtNPs. This assumes a total absorption by the wafer, i.e., all of the light reaching the Si wafer is absorbed. The reflectance of each substrate, i.e., the VA-SiNW array coated with Pt, was calculated using the 2D reflectance monitor localized above the SiNWs, which we refer to as R_SiNWs+PtNPs_. Both, A_wafer_ and R_SiNWs+PtNPs_ were integrated across the 300 to 1200 nm range, normalized by dividing the integrated value by 900, which corresponds to the integrated value of a 100% reflectance over the whole range of wavelength. These values are shown in Figure 5h (termed “Reflection” and “Absorption Si wafer”).

The absorptance in the SiNWs and the Pt catalyst is calculated using eq 2:

2 $$A_{SiNWs+PtNPs}\left(in\%\right)=100-R_{SiNWs+PtNPs}\left(in\%\right)-T_{SiNWs+PtNPs}\left(in\%\right)$$

Because the absorption inside the SiNW is modified in the presence of the Pt, a 3D composition-specific data treatment is necessary to isolate the relative contributions of the SiNW and of the Pt when the SiNW is coated with the Pt catalyst. 3D absorbed power monitors were used to calculate the absorption within the different components, i.e., the SiNW and the Pt. Because of total absorption in the sample, we have R_SiNW+PtNPs_ + A_wafer_ + A_SiNW_ + A_Pt_ = 100%, where A_SiNW_ and A_Pt_ are the absorptance in the SiNW and the Pt, respectively, when the SiNW are coated with Pt. Both, R_SiNW+PtNPs_ and A_wafer_ are known and extracted directly from the 2D monitors. We can thus calculate the sum of the light absorption in the SiNW and the Pt according to eq 3:

3 $$A_{\mathrm{SiNW}}+A_{\mathrm{Pt}}=100\%-R_{\mathrm{SiNW}+\mathrm{PtNPs}}-A_{\mathrm{wafer}}$$

Because the absorbed power data can be used as a relative measure of the light absorption in the SiNW and the Pt when the SiNW is coated with Pt, we calculate the absorptance in the SiNW by applying eq 4:

4 $$A_{SiNW}\left(\mathrm{in \%}\right)=\left(A_{SiNW}+A_{Pt}\right)\times \frac{\int_{300}^{1200}P_{SiNW}\left(\lambda \right)}{\int_{300}^{1200}P_{SiNW}\left(\lambda \right)+\int_{300}^{1200}P_{Pt}\left(\lambda \right)}$$

where $P_{Pt}\left(\lambda \right)$ and $P_{SiNW}\left(\lambda \right)$ are the wavelength dependent absorbed powers in the Pt and the SiNW, respectively. Substituting eq 3 into eq 4 gives eq 5:

5 $$A_{SiNW}\left(\mathrm{in \%}\right)=\left(100\%-R_{SiNW+PtNPS}-A_{wafer}\right)\times \frac{\int_{300}^{1200}P_{SiNW}\left(\lambda \right)}{\int_{300}^{1200}P_{SiNW}\left(\lambda \right)+\int_{300}^{1200}P_{Pt}\left(\lambda \right)}$$

Similarly, we calculate the absorptance in the Pt using eq 6:

6 $$A_{Pt}\left(\mathrm{in \%}\right)=\left(A_{SiNW}+A_{Pt}\right)\times \frac{\int_{300}^{1200}P_{Pt}\left(\lambda \right)}{\int_{300}^{1200}P_{SiNW}\left(\lambda \right)+\int_{300}^{1200}P_{Pt}\left(\lambda \right)}$$

Finally, both A_SiNW_ and A_Pt_ were integrated and normalized, giving the values shown in Figure 5h.

### 5.13 Applied Bias Photon-to-Current Efficiency (ABPE)

The applied bias photon-to-current efficiency (ABPE) was calculated from the LSV curves according to eq 7:

7 $$ABPE=-J_{ph}\times \frac{\left(V_{bias}-V_{H+/H2}\right)}{P_{in}}\times 100$$

where, *J*_*ph*_ (mA/cm^2^) represents the photocurrent density, *V_bias_* (V) corresponds to the bias voltage vs *RHE*, *V*_*H+/H2*_ is the reduction potential of the hydrogen evolution reaction vs *RHE*, i.e., 0 V, and *P*_*in*_ (mW/cm^2^) is the incident light power density which was set to 110 mW/cm^2^ in our photoelectrochemical test. Figure S8 presents the *ABPE* at potentials positive to *V*_*H+/H2*_ for the samples investigated in this work. The maximum *ABPE* for each sample, which corresponds to the maximum photoelectrode conversion efficiency assuming an ideal Faradaic efficiency for the HER of 100%, is presented in Table S1.

## Supplementary Material



## Notes

SI Note I, a complete description of the materials and methods; SEM images of the bare SiNWs and SiNWs after 3DEAL; reflectance spectra of Pt nanoparticles deposited on flat silicon; photograph of a photoelectrode; FDTD simulation results comparing different Pt nanoparticle sizes; comparison of the experimental and simulated reflectance spectra; absorbed power spectra integrated within different regions (i.e., volumes) of the SiNW with and without a Pt ring located in the center of the nanowire (PDF)
