# Supplementary material for: Optimizing Catalyst Location within Nanostructured Photoelectrodes
Source: ACS Appl Mater Interfaces. 2025 Aug 19;17(34):48670–9. doi: 10.1021/acsami.5c11956 (PMC12400269; doi:10.1021/acsami.5c11956)
Supplement: Supplementary file 1 [file am5c11956_si_001.pdf]

Supporting Information

Optimizing Catalyst Location within Nanostructured Photoelectrodes

Amin Farhadi,[1] *Theresa Bartschmid,* [1] *Johannes Menath,*[2] *Nicolas Vogel*[2] *and Gilles R. Bourret*[1,*]

[1] Department of Chemistry and Physics of Materials, University of Salzburg, Jakob Haringerstraße 2a, A-5020 Salzburg, Austria

[2] Institute of Particle Technology, Friedrich-Alexander University Erlangen-Nürnberg, Cauerstrasse 4, 91058 Erlangen, Germany

*E-mail: gilles.bourret@plus.ac.at

Keywords: silicon nanowires, water-splitting, metal-assisted chemical etching, photocathode, lithography

Supporting Information Note I. Modified 3DEAL to pattern SiO_2 and Pt layers

To form electrical contact between the metal catalyst and the SiNW, the standard 3DEAL procedure (see Figure S2) involves the following experimental sequence: Preferential deposition of a thin SiO_2 coating around the SiNWs, electrodeposition of a planar Au/Ni bilayer film, followed by the selective etching of the Au base layer, conformal SiO_2 deposition around the Ni film and the SiNWs, and etching of the Ni film, which generates free-standing horizontal SiO_2 films at defined axial location along the nanowire length [1]. KOH etching of these SiO_2 horizontal films, as well as the thin passivating SiO_2 shell present around the SiNW surface in the patterned region generates patches where the SiNWs are not passivated. These activated regions can then be used to spatioselectively electrochemically deposit the metal catalyst.

Because KOH etches Si faster than SiO_2, the SiNW can be etched locally during this step, and appropriate timing is essential. While the over-etch is not of great concern for large SiNWs with diameter > 700 nm, it becomes critical for the thin 150 nm wide SiNWs used in this work. If the experimental conditions are not tightly controlled, a significant number of the SiNWs can be either not patterned properly, e.g. still coated with SiO_2, or completely etched (Figure S3). This issue was addressed by developing a simplified 3DEAL step sequence (see Figure 1 in the main text). The revised 3DEAL sequence thus prevents the excessive KOH over-etch caused by the non-uniform etching of the horizontal and conformal SiO_2 films used in the original process (Figure S2) [1].

Table S1. J_{SC}, V_{onset} and *ABPE* of the samples studied in this work.

Sample	J_{SC} (mA.cm^{-2})	V_{onset} (V vs *RHE*)	$ABPE_{max}$ (%)	Passivation layer
Bare SiNW	~ 0	-1.15	~ 0	No
$Pt_{uniform}$	4.38	0.18	0.21	No
Pt_{film}	2.00	0.16	0.14	Yes
Pt_{top}	6.12	0.24	0.32	Yes
Pt_{center}	12.6	0.25	0.66	Yes

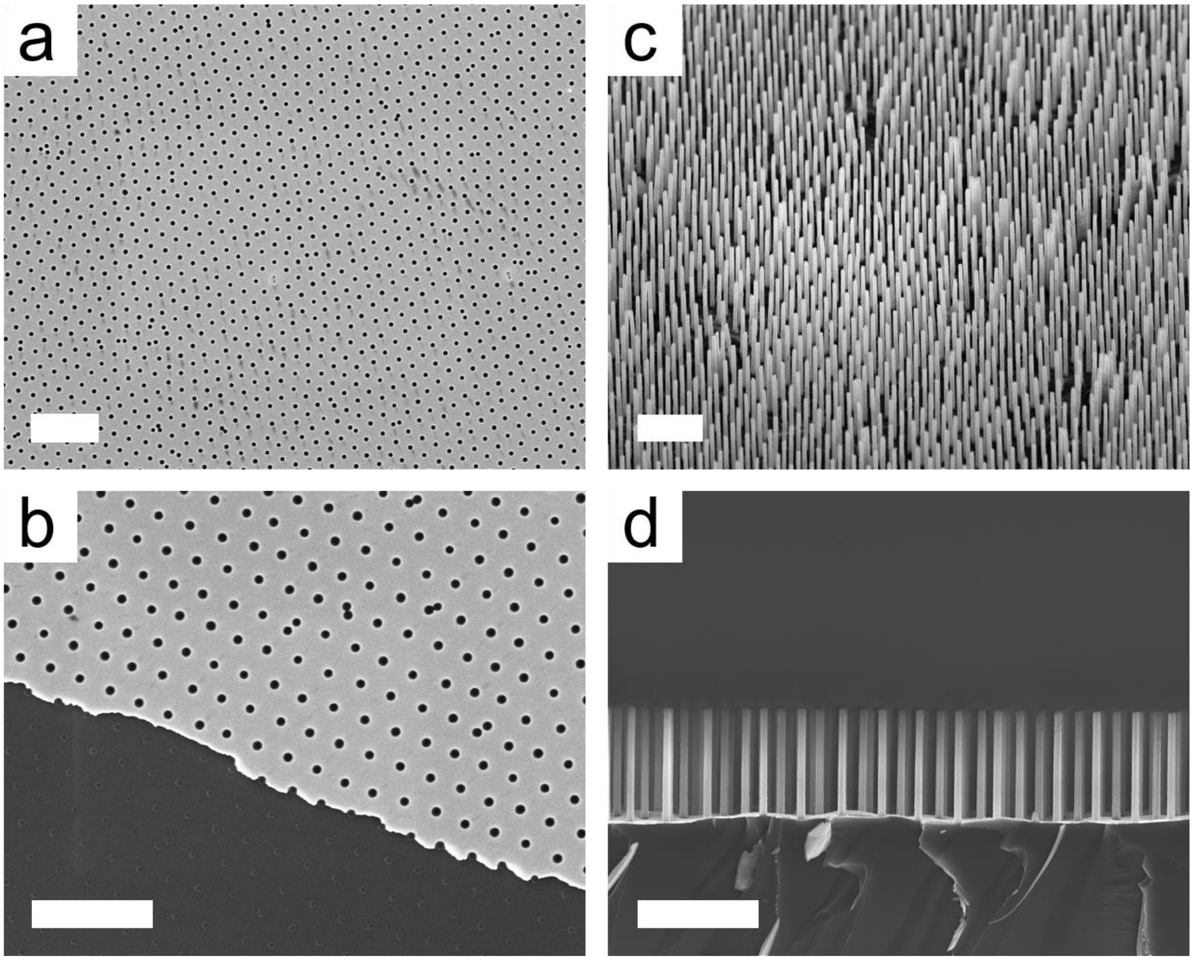

Figure S1. a, b) Top-view scanning electron microscopy (SEM) images of gold nanohole array on the silicon wafer after silica sphere removal; c) tilted SEM view of bare silicon nanowire (SiNW) array; d) cross-sectional SEM image of a bare SiNW array (scale bar: 2 µm).

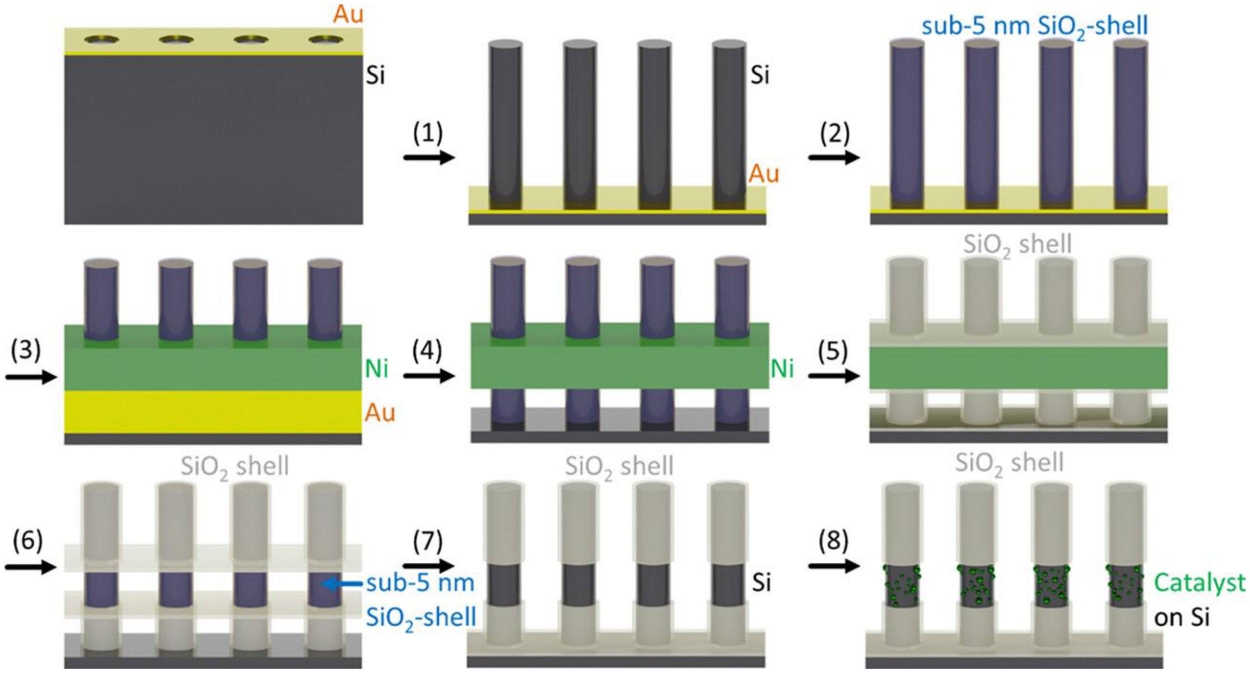

Figure S2. Standard 3DEAL process used to pattern VA-SiNW array with diameters down to 700 nm. (1) Metal-assisted chemical etching (MACE): The gold film etches into the substrate to fabricate silicon nanowire arrays (gray). (2) A protective electrically insulating sub-5 nm SiO_2 shell (dark blue) is deposited on the Si nanowires via sol–gel chemistry. (3) Planar films of gold (yellow) and nickel (green) are sequentially electrochemically grown on the gold base layer. (4) After chemical removal of the gold film, the array is then coated with a conformal SiO_2 film via sol–gel chemistry (white, 5). Subsequent selective etching of the nickel layer (6) leads to silicon nanowire arrays that are patterned with a thick nanostructured SiO_2 layer. Thermal annealing and KOH etching (7) dissolve both the residual horizontal SiO_2 layer deposited on top of the nickel layer and the sub-5 nm SiO_2 shell, yielding SiO_2 passivated silicon nanowire arrays with exposed areas at defined axial positions. Spatioselective electrodeposition of metal particles (green, 8) can now be realized at these defined locations. Caption obtained and figure reprinted from ref [1]. Copyright 2020 American Chemical Society.

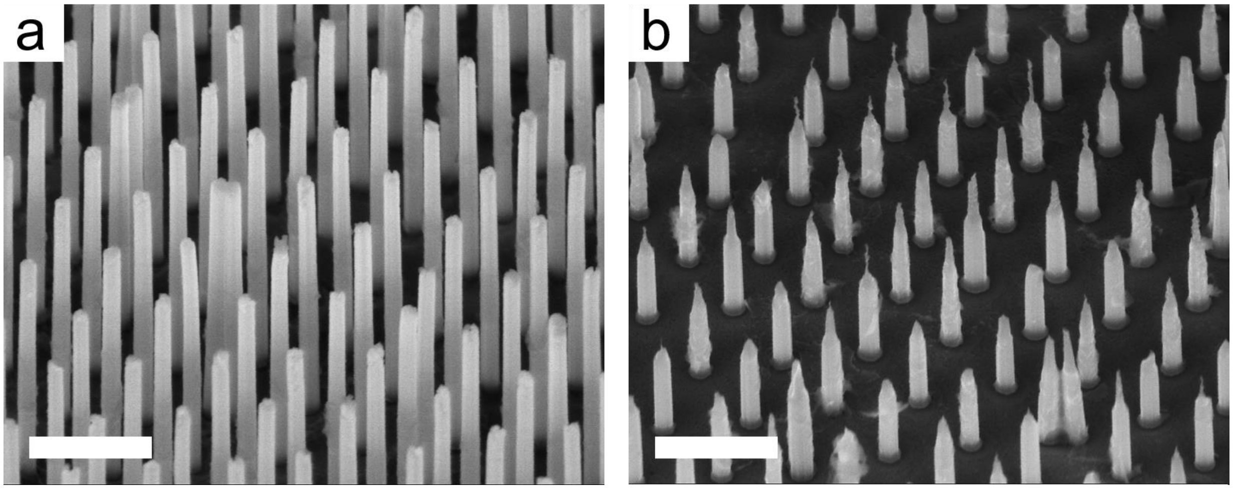

Figure S3. Tilted SEM Images Showing the Effect of KOH Treatment on SiNWs: a) Under-Etched and b) Over-Etched (scale bar: 1 μm).

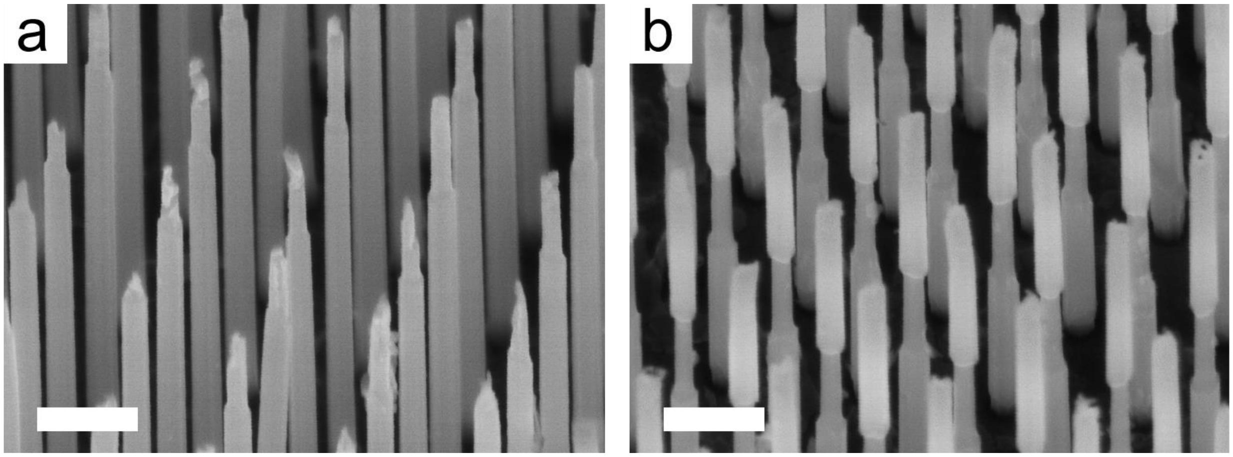

Figure S4. Tilted SEM images of SiO_2-patterned SiNWs, illustrating selective SiO_2 removal: a) top region, b) central region (scale bar: 500 nm).

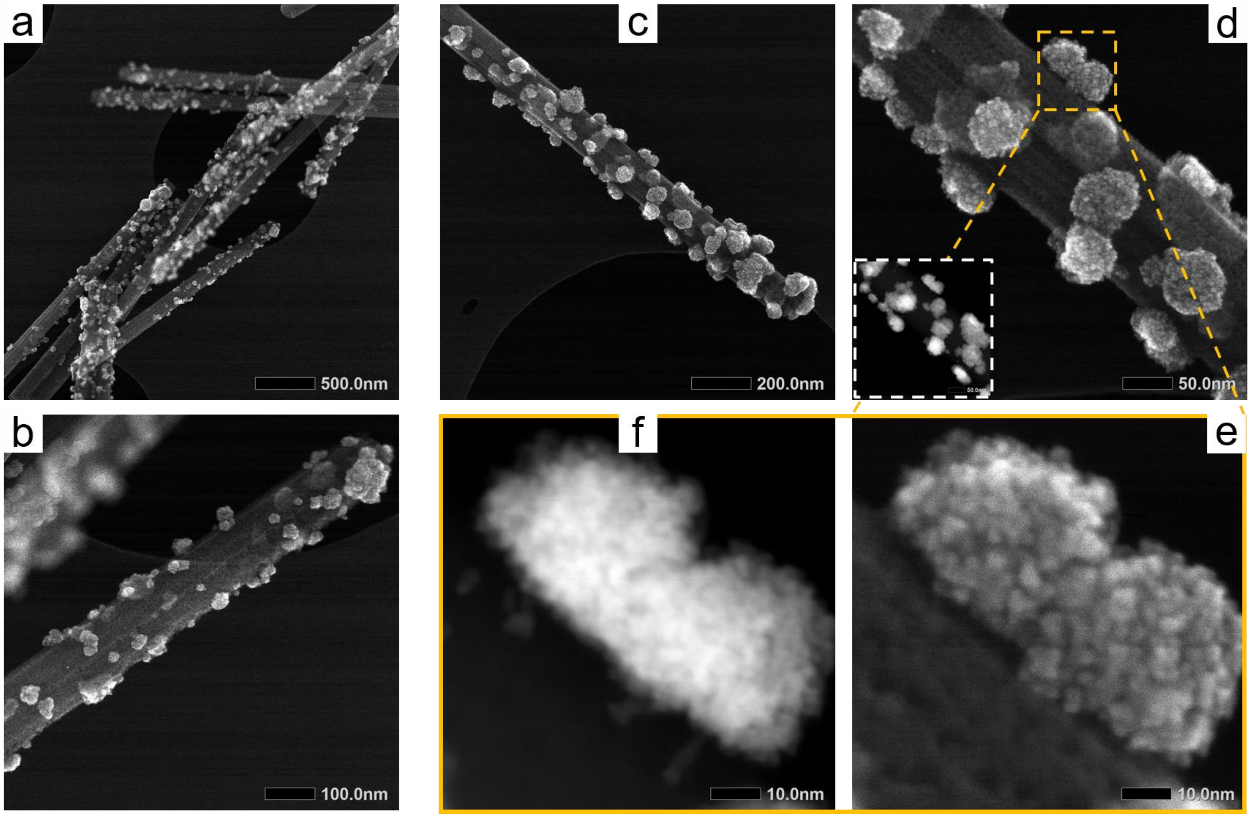

Figure S5. Scanning transmission electron-microscopy (STEM) investigation of SiNWs with a uniform distribution of Pt NPs. a-e) Secondary Electron image illustrating the highly porous nature of the Pt deposits. Inset in d: high angle annular dark-field (HAADF) STEM. f) HAADF-STEM image corresponding to the area depicted in e.

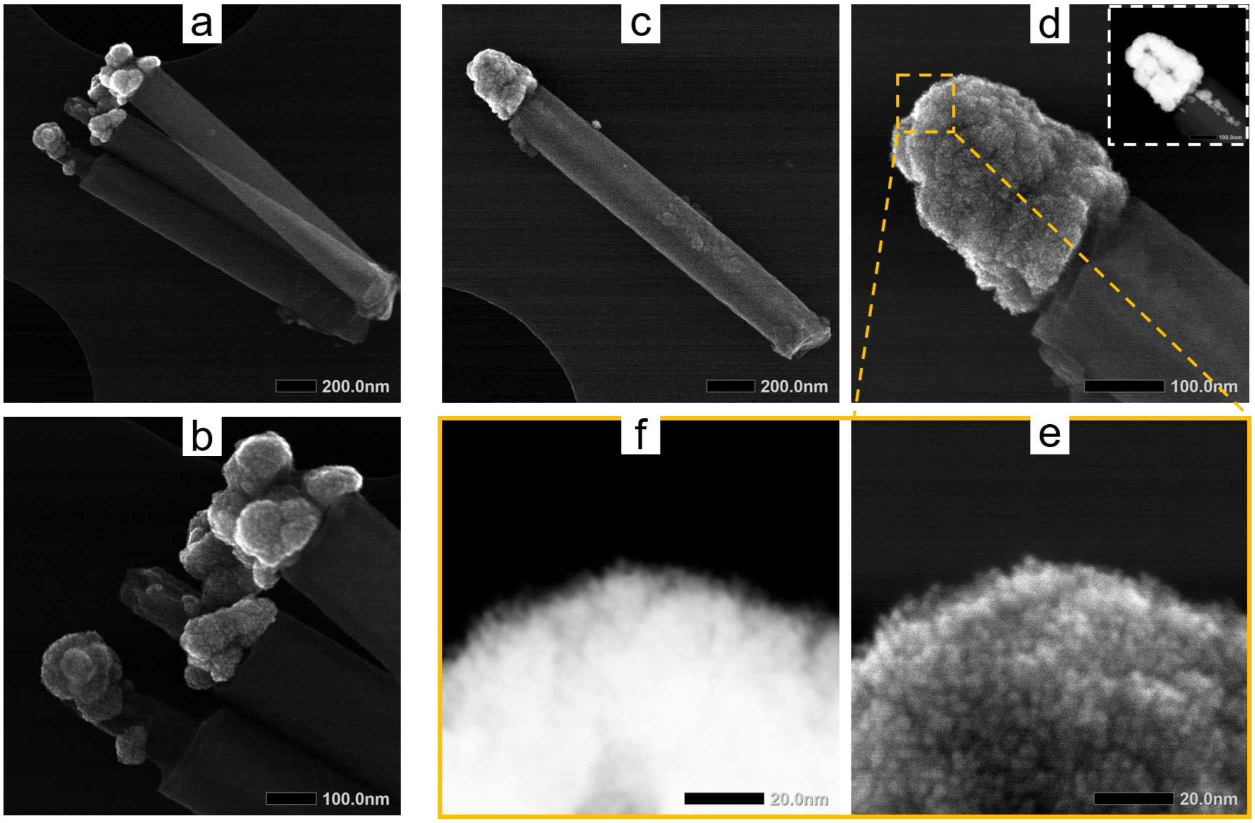

Figure S6. STEM investigation of patterned SiNWs with Pt nanoparticles selectively deposited at the top. a-e) Secondary Electron image illustrating the highly porous nature of the Pt deposits (inset in d: HAADF-STEM). f) HAADF-STEM image corresponding to the area depicted in e.

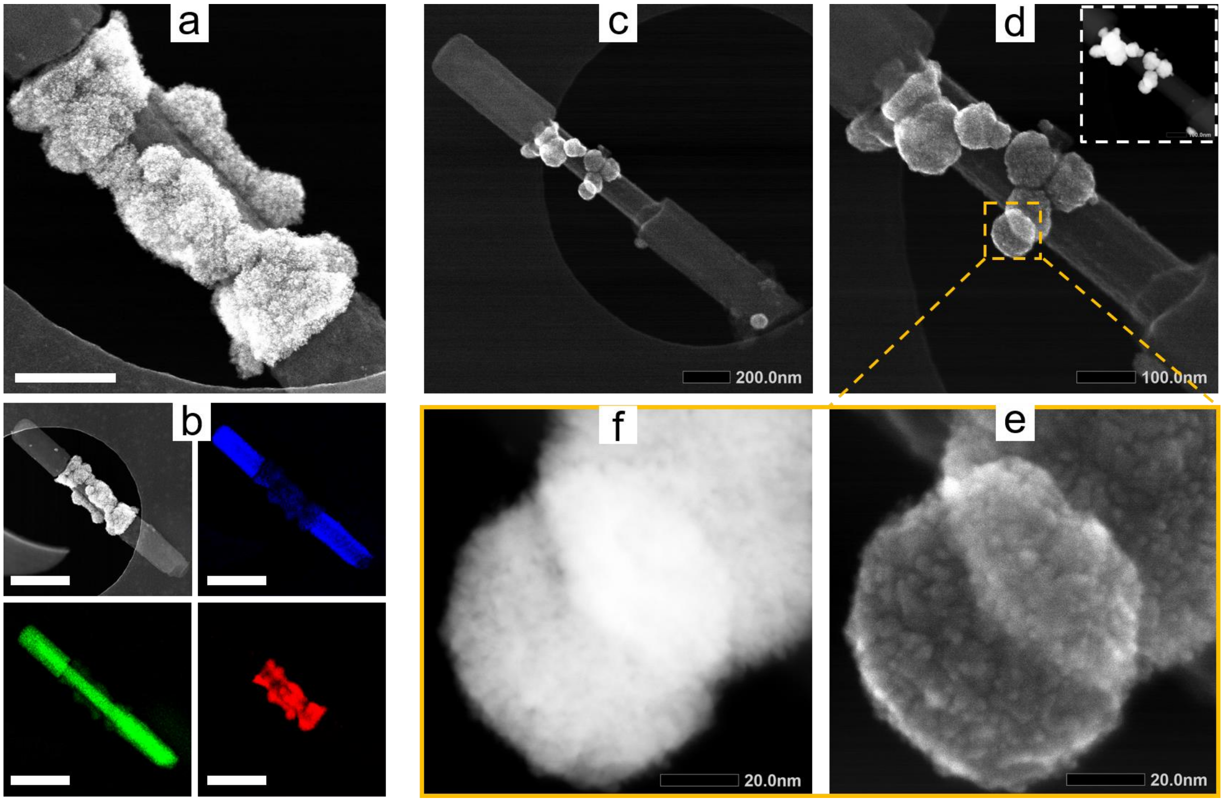

Figure S7. Scanning Transmission Electron Microscopy (STEM) characterization of Patterned SiNWs with central Pt NPs decoration. a) Secondary electron image showing highly porous Pt loads (scale bar: 200 nm). b) Corresponding lower magnification SE image and EDX maps of the same SiNW showing O (blue), Si (green), and Pt (red) distribution (scale bar: 500 nm). c-e) Secondary electron images detailing highly porous Pt loads (inset in d: HAADF-STEM). f) HAADF-STEM image corresponding to the region in e.

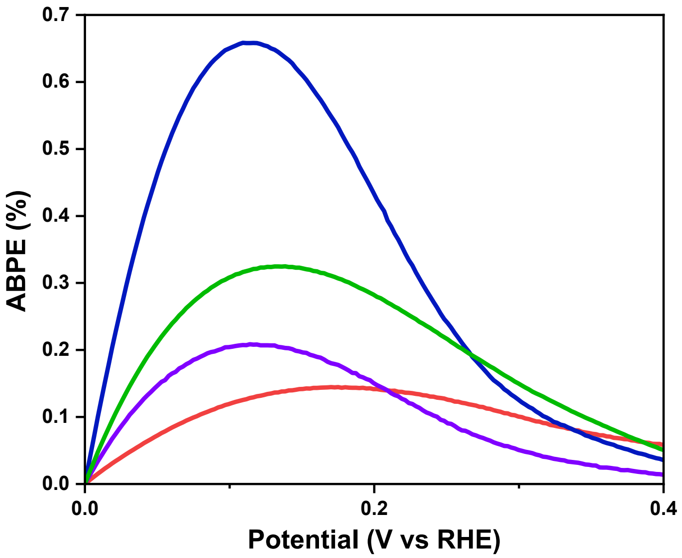

Figure S8. Applied bias photon-to-current efficiency (ABPE) curves for photocathodes with different Pt decoration regimes; Pt_{film} (red solid line), $Pt_{uniform}$ (purple solid line), Pt_{top} (green solid line), and Pt_{center} (solid blue line).

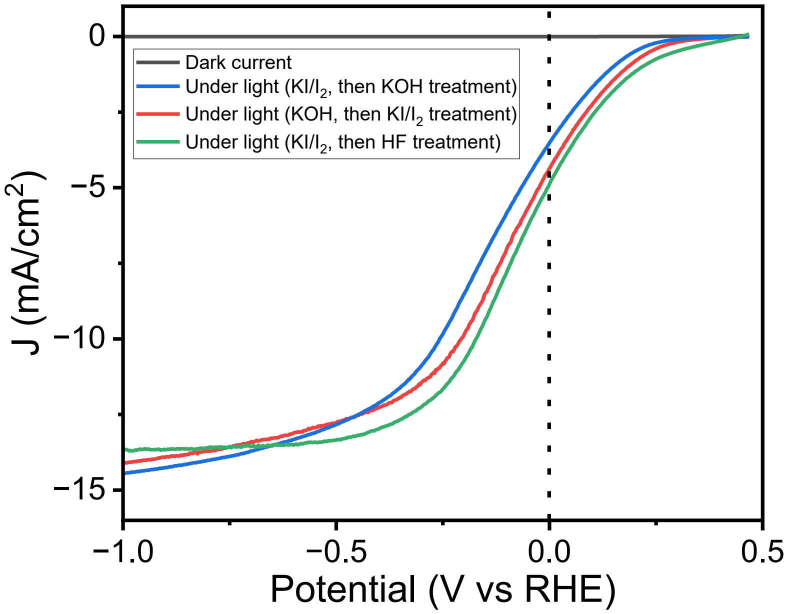

Figure S9. Influence of silicon surface treatment on photocurrent. Linear sweep voltammograms of 3 $Pt_{uniform}$ samples synthesized using three different silicon treatment sequences prior to the Pt electrodeposition: The blue curve corresponds to the sample prepared by first etching the gold film dissolution in KI/I_2, then etching SiO_2 layer in KOH, with a J_{SC} of 3.52 $mA.cm^{-2}$. The red curve corresponds to the sample prepared by first etching SiO_2 with KOH, then the gold film with KI/I_2, with a J_{SC} of 4.38 $mA.cm^{-2}$, which is shown in Figure 2 in the main text. The green curve corresponds to the sample prepared by first etching the gold film in KI/I_2, followed by HF etching, with a J_{SC} of 4.90 $mA.cm^{-2}$. Overall, the values are quite close. This suggests that the Si-Pt interface quality cannot, by itself, explain the large differences we have measured in J_{SC} between the samples measured and discussed in the main text.

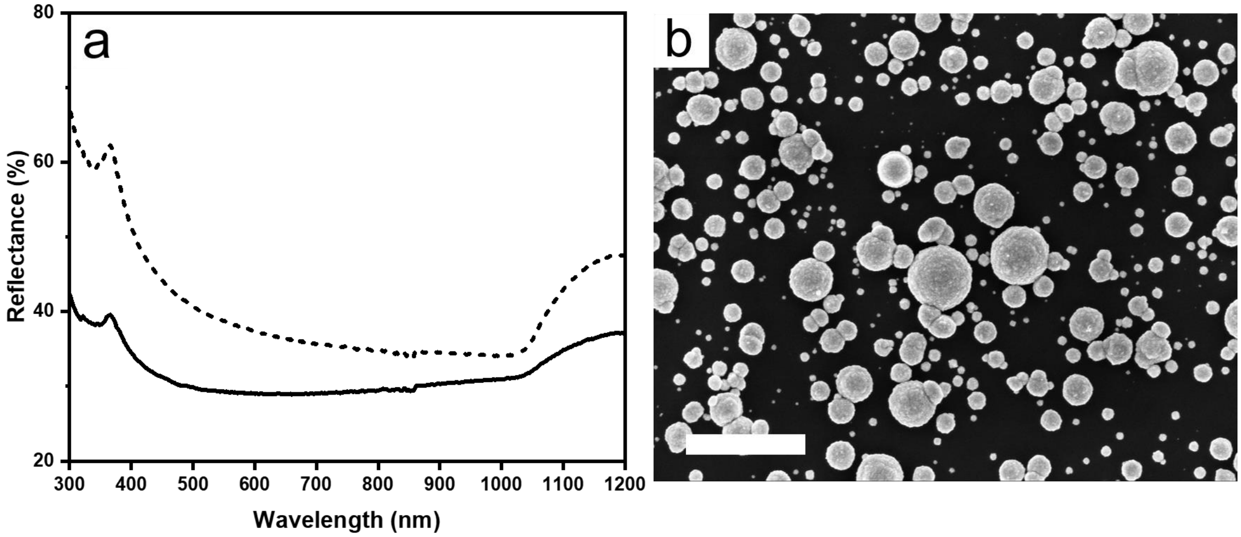

Figure S10. a) Reflectance spectra of bare flat Si (dashed line) and Pt-decorated flat Si (solid line). b) Top-view SEM image of the Pt-decorated Si substrate (scale bar: 1 µm).

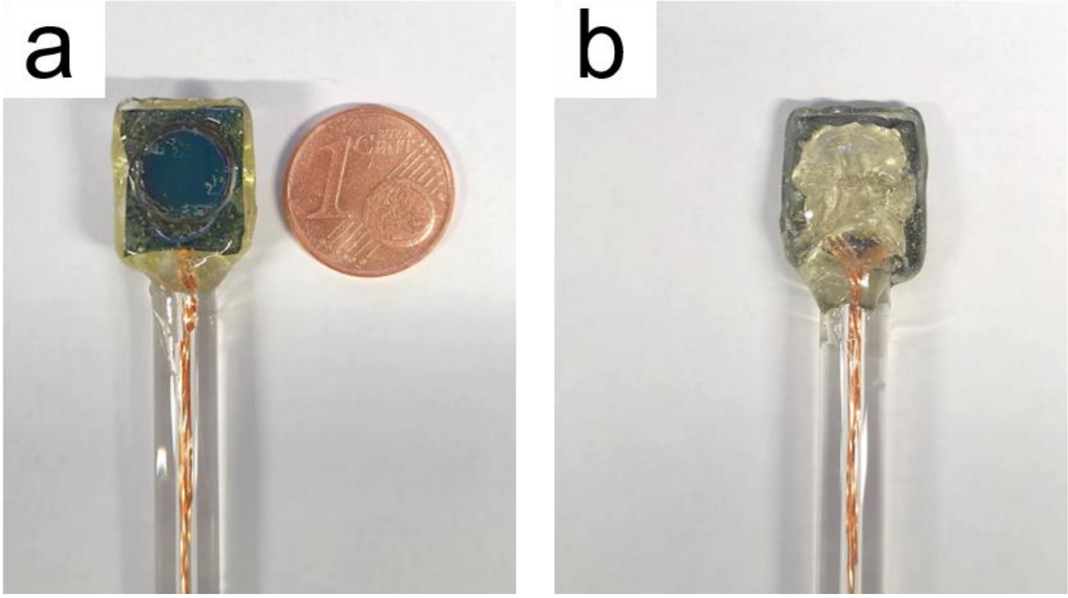

Figure S11. Photograph of the photocathode employed in photoelectrochemical measurements: a) Front view, illustrating the exposed Pt-decorated region and the epoxy seal; b) Back view, depicting the connection to copper wires via Ga-In eutectic and silver epoxy.

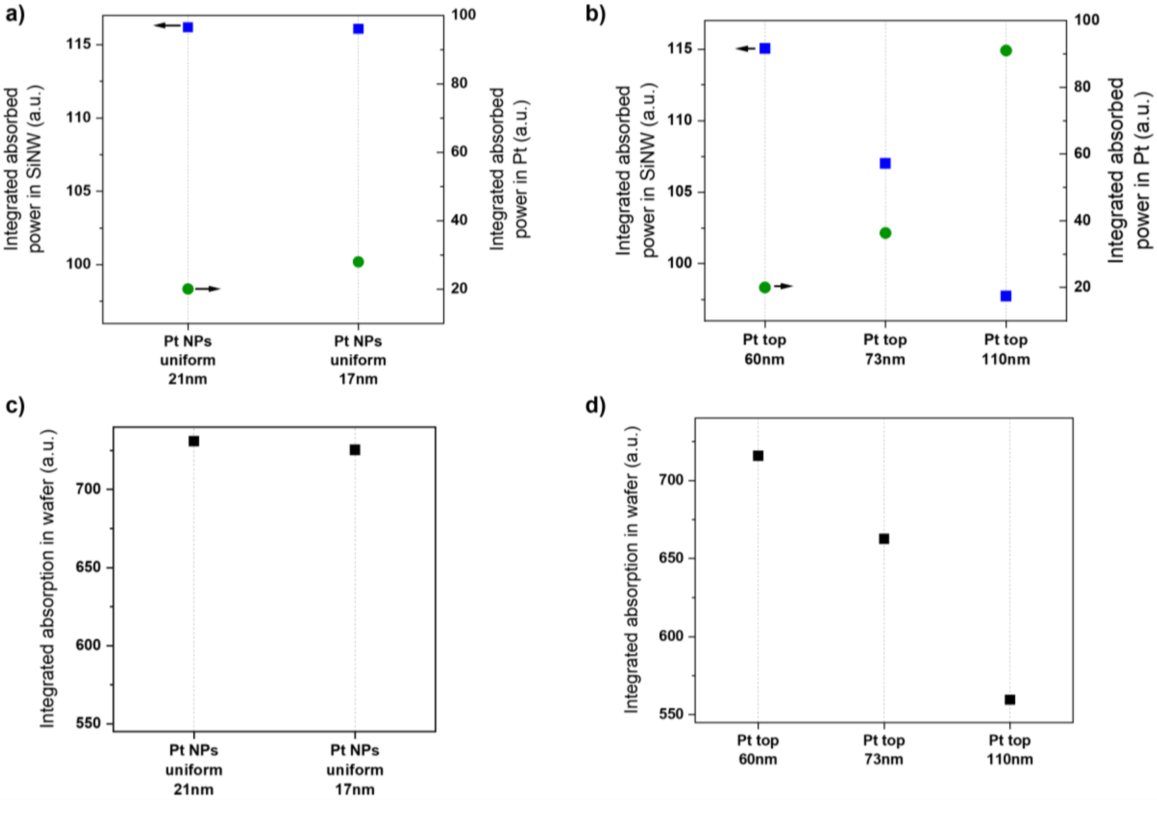

Figure S12. a, b) Effect of PtNP size on integrated absorbed power in SiNWs (blue squares) and Pt (green circles); c, d) Effect of PtNP size on integrated absorption in the silicon wafer. a, c) Uniform PtNP distribution along the SiNW axis. b, d) Single PtNP at the SiNW top. PtNP diameter is shown on the x-axis.

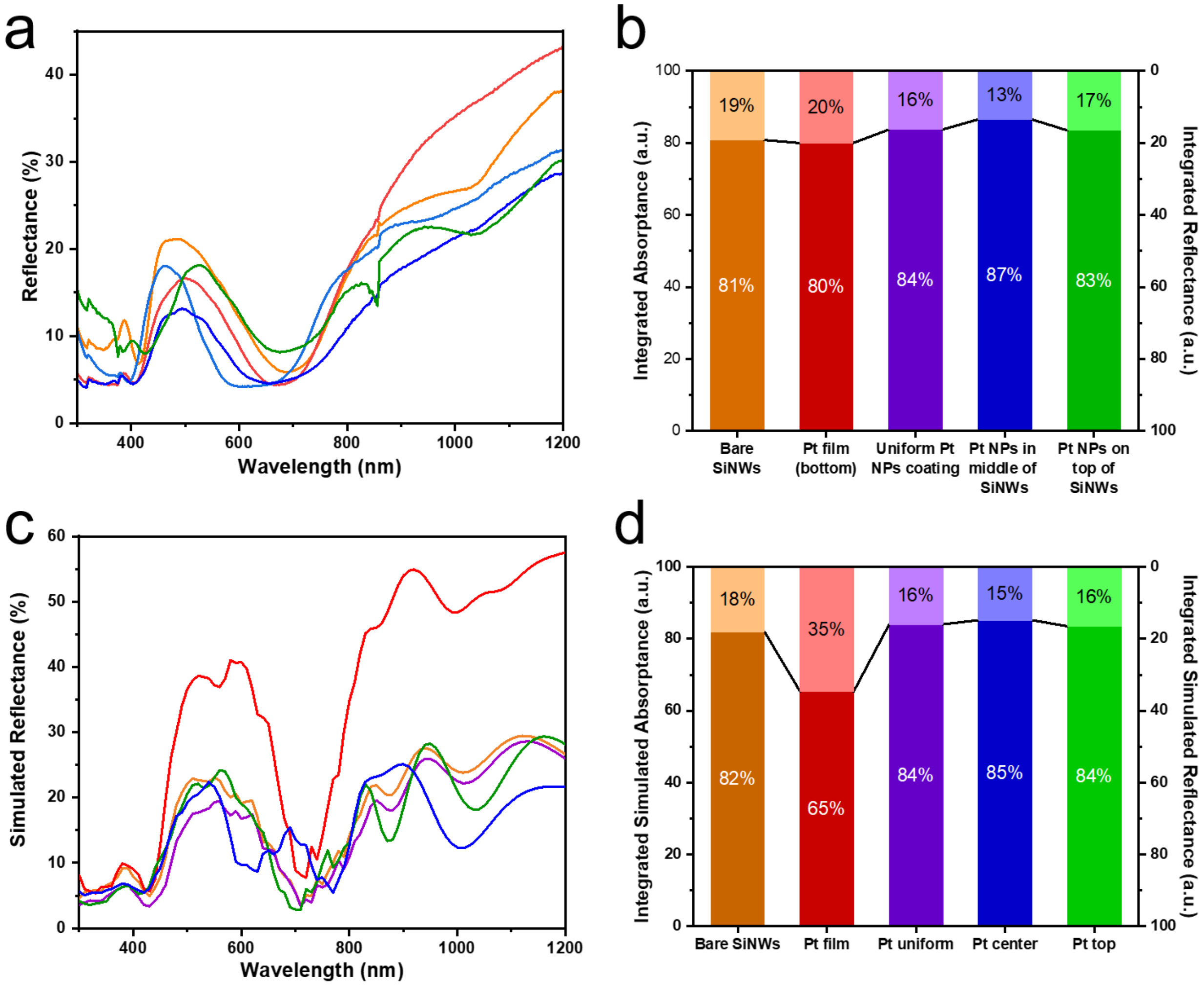

Figure S13. a) Measured reflectance spectra within the 300–1200 nm wavelength range for: bare SiNWs (orange), SiNWs with Pt film at the bottom (red), SiNWs with uniform Pt NPs decoration (purple), SiNWs with Pt at the top (green), and SiNWs with Pt in the center (blue). b) Corresponding normalized integrated values of absorbance and reflectance. c) Simulated reflectance spectra within the 300–1200 nm wavelength range for the same Pt-decorated SiNWs configurations, and d) Corresponding normalized integrated values of absorptance (the lower stacks) and reflectance (the upper stacks).

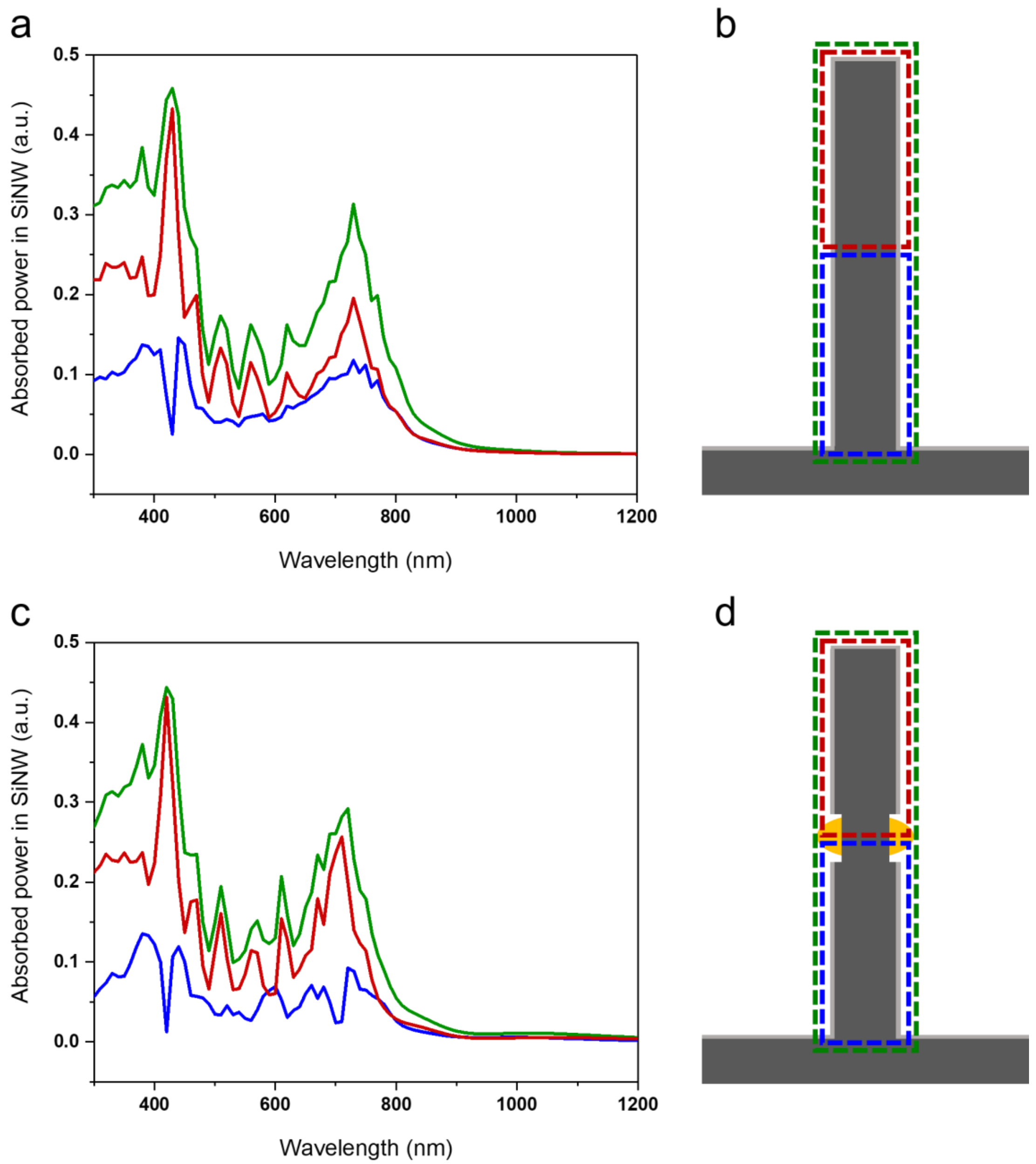

Figure S14. Absorbed power the SiNW (green) split by the respective contributions of lower (blue) and upper (red) half of the SiNW. (a,b) Bare SiNW. (c, d) Sample where the Pt is present as a Pt shell located in the around the center of the SiNW (sample Pt_{center}).

References

(1) Wendisch, F. J.; Abazari, M.; Werner, V.; Barb, H.; Rey, M.; Goerlitzer, E. S. A.; Vogel, N.; Mahdavi, H.; Bourret, G. R. Spatioselective Deposition of Passivating and Electrocatalytic Layers on Silicon Nanowire Arrays. *ACS Appl. Mater. Interfaces* **2020**, *12* (47), 52581-52587.
